# Spatial Pattern and Decomposition Analysis of the Educational Status Gap in Factors Associated with Risky Sexual Behavior Among Women with Disabilities in Ten African Countries

**DOI:** 10.1007/s10508-025-03279-z

**Published:** 2025-11-13

**Authors:** Clifford Odimegwu, Obasanjo Bolarinwa, Aliu Mohammed, Ezra Gayawan

**Affiliations:** 1 Demography and Population Studies Programme, Schools of Public Health and Social Sciences, University of the Witwatersrand, Johannesburg, South Africa; 2 Department of Business, Management and Health, York St John University, London, E14 2BA UK; 3 Department of Health, Physical Education and Recreation, University of Cape Coast, Cape Coast, Ghana; 4 Department of Statistics, Federal University of Technology, Akure, Akure, Ondo State Nigeria

**Keywords:** Spatial pattern, Decomposition analysis, Educational status, Risky sexual behavior, Women with disabilities, Demographic Health Survey

## Abstract

Despite significant improvements in access to sexual and reproductive health services worldwide, many people with disabilities, particularly in low-income settings, remain vulnerable to risky sexual behaviors, predisposing them to sexual and reproductive health problems. This study examined the spatial patterns and performed a decomposition analysis of the educational status disparity in factors associated with risky sexual behavior among women with disabilities in 10 African countries. We utilized the latest secondary dataset with a disability module from demographic health surveys conducted in these countries between 2010 and 2022, including a sample size of 16,517 women with disabilities. Spatial analysis was employed to reveal the patterns of risky sexual behavior, while multivariable Blinder–Oaxaca decomposition regression analysis examined the disparity between educational status and risky sexual behavior. The analysis accounted for the complex survey design and results were presented using percentages and adjusted coefficients. The spatial pattern of risky sexual behavior among women with disabilities varied widely across the 10 African countries studied, with proportion ranging from 20 to 80%. Kenya leads with the highest prevalence at 94%, followed by Mali at 90%, while Mauritania reported no occurrence at 0%. Most of the disparity (81.93%) in risky sexual behavior related to educational status among women with disabilities was due to differences in coefficients, with the remaining 18.07% attributed to differences in characteristics. The findings indicate that women with disabilities, whether with formal or informal education, are highly exposed to risky sexual behavior in Africa. Therefore, targeted interventions are needed to minimize risky sexual behavior among women with disabilities in these countries.

## Introduction

Despite the significant improvement in access to sexual and reproductive health services worldwide (Liang et al., 2019; World Health Organization, 2016), many people with disabilities, especially in low- and middle-income countries, continue to be marginalized and denied their sexual and reproductive rights (Addlakha et al., 2017; Casebolt, 2020). Often, interventions to address sexual and reproductive health issues do not target people with disabilities, although they constitute about 16% of the global population (Ganle et al., 2020; World Health Organization [WHO], 2022). This increases the vulnerability of people with disabilities to risky sexual behaviors and thus predisposes them to sexual and reproductive health problems, including unplanned pregnancies, HIV and other sexually transmitted infections [STIs] (De-Beaudrap et al., 2014; Groce et al., 2009). Besides, people with disabilities, particularly women with disabilities in sub-Saharan Africa, are highly exposed to sexual violence, exploitation, and abuse (Odimegwu et al., 2025; Ortoleva & Lewis, 2012; Violence Against Women & Girls, 2019), thus compromising their safety and sexual and reproductive health outcomes.

Risky sexual behavior is generally described as sexual activity that exposes the individual to the risk of contracting HIV and other STIs (Chawla & Sarkar, 2019). It includes having unprotected sexual intercourse (e.g., condomless sex), having multiple sexual partners, having sex under the influence of alcohol or drugs, and being physically forced to have sexual intercourse early age (Eaton et al., 2012), having a risky sexual partner (e.g., a partner with multiple sexual partners), having unprotected mouth-to-genital sex, and engaging in paid sex (De-Beaudrap et al., 2017). Aside from increasing the risk of unintended pregnancies, unsafe abortion (Alhusen et al., 2021) and STIs, including HIV (De-Beaudrap et al., 2014), risky sexual behaviors are also associated with some forms of cancers, such as cervical, vulval, vaginal, and oropharyngeal cancers (Grulich et al., 2010).

While risky sexual behaviors are common among the general population in Africa (Uchudi et al., 2012), particularly among adolescents and young women (Birdthistle et al., 2019), evidence suggests that women with disabilities have the greatest exposure to risky sexual behaviors, which increase their risk for HIV and other STIs (De-Beaudrap et al., 2014; Groce et al., 2009). For example, in Ethiopia, Kassa et al. (2014) found that people with disabilities were more likely to have multiple sexual partners, casual sexual partners, and commercial sexual partners. In Cameroon, De-Beaudrap et al. (2017) reported a higher prevalence of HIV infection among women with disabilities than those without disabilities, which was attributed to the increased involvement of women with disabilities in paid sexual relationships and sexual violence. Factors associated with increased involvement of women with disabilities in risky sexual behaviors include having limited knowledge of the prevention of HIV and STIs, having low life skills, and increased alcohol abuse (Bukuluki et al., 2023). Others include social inequality, poverty, limited access to sexual and reproductive healthcare (Parekh et al., 2023), and lack of protection for the sexual and reproductive rights of women with disabilities (Addlakha et al., 2017).

Meanwhile, despite being as sexually active as their peers without disabilities (Aderemi et al., 2013; Haynes et al., 2018) and having greater exposure to risky sexual behaviors (Groce et al., 2009), women with disabilities receive limited sexual and reproductive health education compared to their peers without disabilities (Namkung et al., 2021). This limits the knowledge of women with disabilities on safe sex practices, including the use of condoms and other contraceptives (Horner-Johnson et al., 2019). Besides, women with disabilities face physical, communication and attitudinal barriers in accessing sexual and reproductive health services (Mac-Seing et al., 2020), which further compounds their risk for sexual and reproductive health problems.

Even though women with disabilities have the greatest risk for HIV infection due to their increased exposure to risky sexual behaviors (Groce et al., 2013), studies on risky sexual behavior in Africa have predominantly focused on male and female adolescents, young women (Page & Hall, 2009; Ssewanyana et al., 2018), female sex workers and their clients (Dhana et al., 2014; Scorgie et al., 2012), and men who have sex with men (Fiorentino et al., 2024; Sandfort et al., 2017). Thus, data are scarce on risky sexual behaviors of women with disabilities in Africa. Perhaps this contributes to the limited availability of targeted interventions to address or prevent risky sexual behaviors among women with disabilities in Africa. Despite the importance of education on sexuality and sexual risk behavior of women with disabilities (Alamrew et al., 2014), little is known about the educational status disparity in factors associated with risky sexual behaviors among women with disabilities in Africa. To the best of our knowledge, no previous study has investigated the phenomenon in Africa. Knowing the educational status disparity in factors that influence risky sexual behavior among women with disabilities in multiple countries in Africa could help in designing and implementing targeted interventions that address the phenomenon among the most vulnerable population of women with disabilities.

## Method

### Participants

This study utilized the most recent standardized data from the Demographic Health Survey (DHS) conducted across ten African nations. The DHS is a national survey implemented in over 90 countries to collect essential health indicators among individuals aged 15–49 (Aliaga & Ren, 2006). Data for this research were obtained from the recode files of women and households, all of which are publicly accessible upon request (Corsi et al., 2012). The women's recode file included data on maternal, sexual, and reproductive health, while disability information was drawn from the household recode file. Only countries with available disability modules and complete responses for the study variable of interest were included in the study, all respondents without responses for sexual behavior variables were dropped (DHS Program, 2023). The DHS utilizes a two-stage sampling process: first, primary survey units are selected and then participants are randomly chosen from clusters within each country. For this study, women aged 15–49 with disabilities were eligible to participate in the disability module. Specifically, one woman with a disability from every third selected household was included (Corsi et al., 2012; Ties Boerma & Sommerfelt, 1993). Participants were identified as having one or more disabilities based on the Washington Group Short Set of Disability Questions, which focuses on difficulties with seeing, hearing, speaking, and walking (Casebolt, 2021; DHS Program, 2017). The analysis included 16,157 weighted women with disabilities who provided complete data on educational status, risky sexual behavior, and relevant covariates.

Table 1 details the sample sizes for various countries in Africa. The DHS is recognized as a reliable secondary dataset and has been extensively used in research on sexual and reproductive health, including risky sexual behavior in Africa (Bolarinwa et al., 2023; Odimegwu et al., 2016). Data for this study were accessed from the DHS website on February 23, 2024, following a request via https://dhsprogram.com/data/available-datasets.cfm.

**Table 1:** Distribution of weighted eligible Demographic Health Surveys in 10 African countries

S. no	Country	Survey year	Sample size	Sample size percentage
1	Congo DR	2013/2014	991	6.13
2	Chad	2014/2015	1553	9.61
3	Uganda	2015/2016	4168	25.80
4	Malawi	2015/2016	1499	9.28
5	South Africa	2015/2016	836	5.17
6	Mali	2017/2018	947	5.86
7	Nigeria	2017/2018	629	3.89
8	Rwanda	2019/2020	1694	10.48
9	Mauritania	2019/2022	2436	15.08
10	Kenya	2021/2022	1404	8.69

### Measures

#### Study Population Variable

The study population for this study was women with disabilities. Women with disabilities were identified using the DHS disability module questionnaire, which asked the household head about the disability status of women of reproductive age. These questions followed the Washington Group's short set of disability questions, addressing difficulties in seeing, walking, hearing, remembering, communicating, and self-care for women aged 15–49. The response options were "No difficulty," "Some difficulty," "A lot of difficulty," and "Cannot do it at all." This study focused specifically on difficulties in seeing, hearing, speaking, and walking. Respondents with “No difficulty” responses were excluded, and all women with at least one functional difficulty in these areas across ten African countries were included in this study as women with disabilities (DHS Program, 2017).

#### Outcome Variable

The primary outcome of this study was risky sexual behavior, and this was defined by two questions on the number of sex partners and condom use in the last sex act by women with disabilities. Responses on the number of sex partners were reported in numeric. Women with disabilities who reported 0 to 1 partner were categorized as “0,” representing no multiple partners, while women with disabilities who had more than one sex partner were categorized as “1,” representing multiple sexual partners (Simelane et al., 2021). Similarly, women with disabilities who reported having used condoms in the last sex were categorized as “0” for having condom sex, while those who reported not using condoms in their most recent sex were categorized as “1” for having condomless sex (Bolarinwa et al., 2022).

To derive the risky sexual behavior variable, a composite was constructed using two labels by combining the women with disabilities who responded “no multiple partners and having condom sex” for both numbers of sex partners and condom use in the last sex, respectively, as “0” to represent “no” while those who were categorized as “having multiple partners and condomless sex” were combined to represent “yes” (Odimegwu et al., 2016).

#### Key Independent Variable

The key independent variable for this study was educational status, which was categorized as “0” for informal education to represent those who had no educational status, while women with disabilities with at least primary education and above were categorized as “1” to represent those with formal education. In DHS, educational status is operationalized as formal and informal because those who reported not to have had education were not illiterate but had one informal education such as home teaching, which may enable them to be able to read and write, which was captured as “no education” (Aliaga & Ren, 2006).

### Covariates

The covariates for the logistic regression analysis were selected based on their relevance in the existing literature (Bolarinwa et al., 2022; Odimegwu et al., 2016; Simelane et al., 2021). These covariates include age, marital status, wealth index, mass media exposure, sexual health knowledge, knowledge of contraceptive methods, place of residence, community literacy level, community socioeconomic level, and community knowledge of modern methods.

Women with disabilities were categorized into three age groups: 15–24, 25–34, and 35 and above. Marital status was divided into never married, currently married, and ever married. The wealth index was divided into five categories: poorest, poorer, middle, richer, and richest. According to DHS, mass media refers to exposure to newspapers/magazines, radio, or television. It is measured by how often individuals access these media, typically at least once a week as such; mass media exposure was categorized as either no exposure or exposure to mass media. Sexual health knowledge and knowledge of contraceptive methods were categorized as poor, moderate, and good. The place of residence was categorized into rural and urban. Additionally, community literacy level and community socioeconomic level were classified as low, medium, and high (Aliaga & Ren, 2006; Bolarinwa et al., 2022; Odimegwu et al., 2016; Simelane et al., 2021).

### Statistical Analyses

The data were analyzed using both spatial and multilevel decomposition analysis.

#### Spatial Analysis

Geospatial maps were generated using a geostatistical model that treated observations as binary indicators governed by a binomial probability distribution with success probability *p*. This probability was linked to a random spatial term via a logit link function (Barrero et al., 2011). The spatial random term captured the spatial distribution of risky sexual behavior data, assuming spatial dependence across locations. This term was modeled with a Gaussian distribution, having a zero mean and a spatially structured covariance matrix (Gómez-Rubio et al., 2020).

The model is defined as:

$$ {\text{logit}}\left( {{\text{pi}}} \right) \, = \, \beta_{0} + \, u\left( {{\text{si}}} \right), $$

where pi is the probability that a woman with a disability is at location i engages in risky sexual behavior, β_0_ is the intercept, and *u*(si) is a spatial Gaussian random field with a Matérn covariance structure.

The geostatistical inference was performed using stochastic partial differential equations, which represented the continuous spatial random process as a discretely indexed Gaussian Markov random field (Barrero et al., 2011). This method enabled a Bayesian approximation through the integrated nested Laplace approximation (Barrero et al., 2011; Gómez-Rubio et al., 2020). The spatial analysis was performed using R software version 4.4.1 (R Foundation for Statistical Computing, Vienna, Austria).

#### Multivariate Decomposition Analysis

A pooled analysis of the proportion of risky sexual behavior among women with disabilities was conducted, including a tabulation based on educational status and selected covariates. This was followed by a chi-square (*χ*^*2*^) test to examine the association between risky sexual behavior and selected covariates across different residential areas (Zhao et al., 1995). The prevalence of risky sexual behavior among women with disabilities in ten African countries was illustrated using graphs. For inferential analysis, multivariable logistic regression was used to identify predictors of risky sexual behavior, focusing on the key independent variable and covariates (Reichenheim & Coutinho, 2010). Additionally, a multivariate nonlinear decomposition model, similar to the Fairlie and Blinder–Oaxaca methods, was employed to decompose the disparity in risky sexual behavior based on educational status (Rahimi & Hashemi Nazari, 2021). This technique assessed the variation in risky sexual behavior between informal and formal education of women with disabilities and identified the contribution of each covariate to the observed differences in characteristics (E) and coefficients (C) (Rahimi & Hashemi Nazari, 2021). The data were weighted and adjusted for the complex survey design, with the variance inflation factor indicating no evidence of multicollinearity. All analyses were conducted using Stata software version 17.0 (Stata Corporation, College Station, TX, USA).

## Results

### Spatial Pattern of Risky Sexual Behavior

**Fig. 1:**
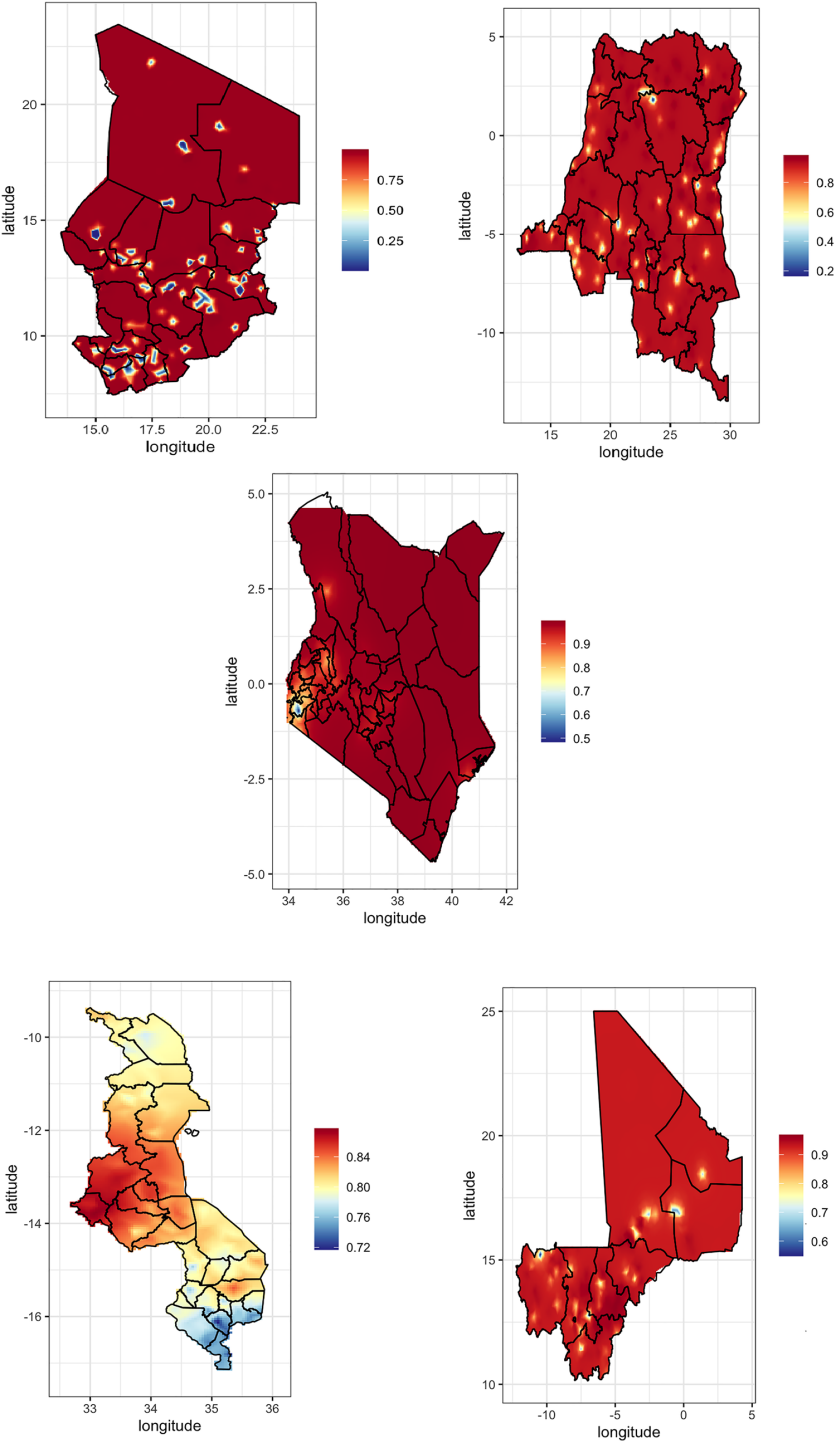
Spatial pattern of risky sexual behavior among women with disabilities in Chad, DR Congo, Kenya, Malawi, and Mali (2013–2022)

**Fig. 2:**
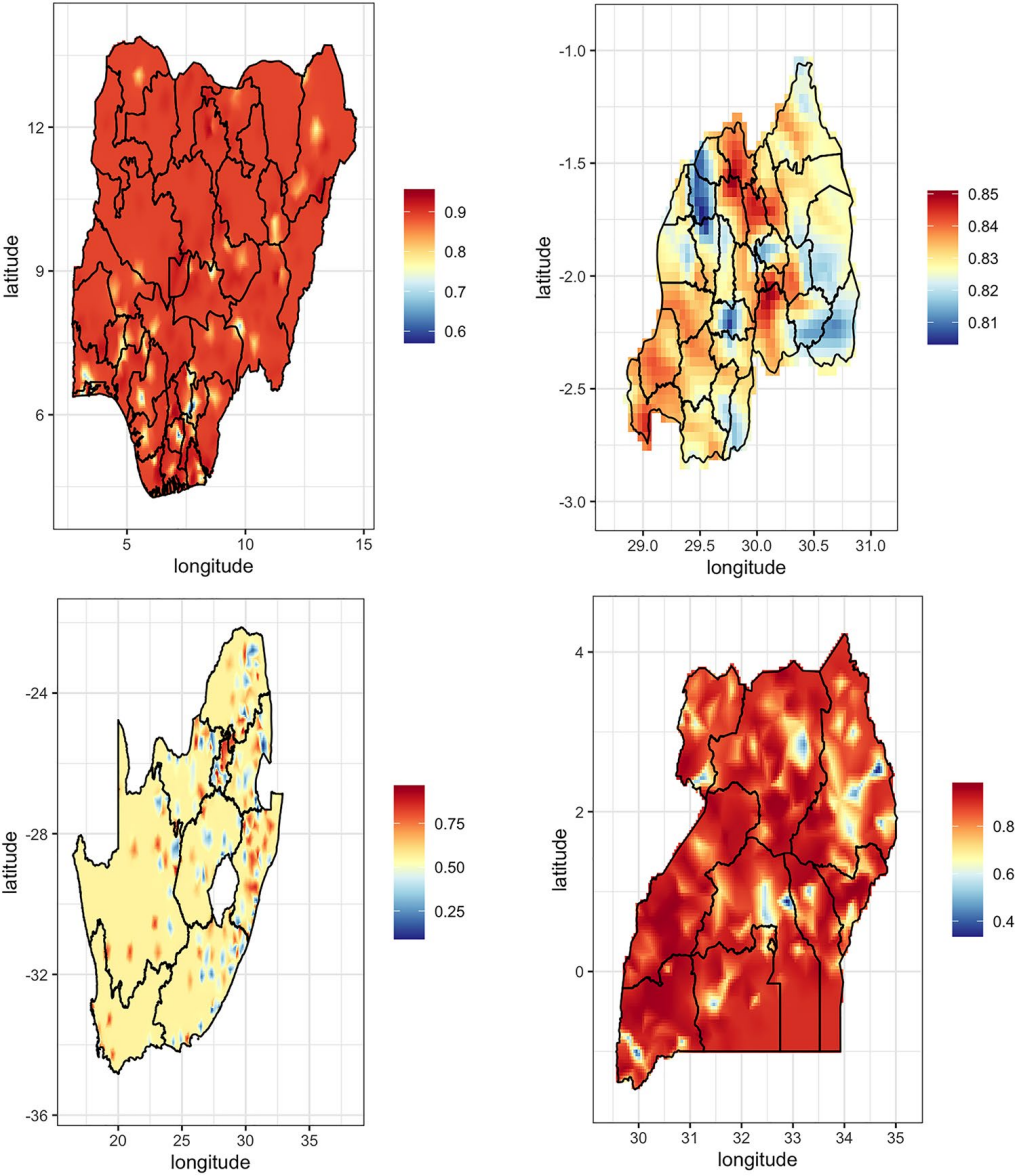
Spatial pattern of risky sexual behavior among women with disabilities in Nigeria, Rwanda, South Africa, and Uganda (2013–2022). The Mauritania map was excluded due to no variation

The maps in Figs. 1 and 2 illustrate the spatial variation in the prevalence of risky sexual behavior among women with disabilities across various African countries, with red indicating higher proportions (up to approximately 75%) and blue indicating lower proportions (around 25%).

Chad, Malawi, and Rwanda show similar patterns, with central and southern regions having high prevalence proportions of 70–80%, while northern areas display much lower proportions of 25–40%. The Democratic Republic of Congo and Uganda also exhibit high-risk sexual behavior in central regions, with proportions of 60–80%, and lower proportions in peripheral areas, around 30–40%. Kenya shows a concentration of higher proportions in the western region (around 50%), but overall lower proportions of 20–30% across the country.

Mali and Mauritania display generally lower risk sexual behavior, mostly around 10–20%, except for a few areas in Mali where proportions rise to 30–40%. Rwanda and South Africa exhibit scattered patterns: Rwanda's higher proportions are approximately 40–50%, with lower proportions at 10–20% in other areas; South Africa's central regions display proportions of 60–70%, while large parts of the country show lower prevalence around 20–30% (Figs. 1 and 2).

Table 2 presents the mean prevalence of risky sexual behavior among women with disabilities across nine countries. Kenya (0.98), Chad (0.95), Mali (0.92), Congo (0.91), and Nigeria (0.89) recorded the highest prevalence, while South Africa reported the lowest (0.56). Wider credible intervals in countries like Congo and South Africa indicate greater uncertainty. The overall pooled prevalence was 2.50 (95% CI 1.91–3.09).

**Table 2:** Mean prevalence of risky sexual behavior among women with disabilities across countries, with credible intervals

Country	Prevalence	Lower CI	Upper CI
Chad	0.95	0.07	0.99
Congo	0.91	0.34	0.99
Kenya	0.98	0.89	0.99
Malawi	0.81	0.71	0.89
Mali	0.92	0.61	0.99
Nigeria	0.89	0.64	0.91
Rwanda	0.83	0.76	0.88
South Africa	0.56	0.15	0.90
Uganda	0.87	0.64	0.96
Overall	2.50	1.91	3.09

### Weighted Country-Level Prevalence of Risky Sexual Behavior and Educational Status

**Fig. 3:**
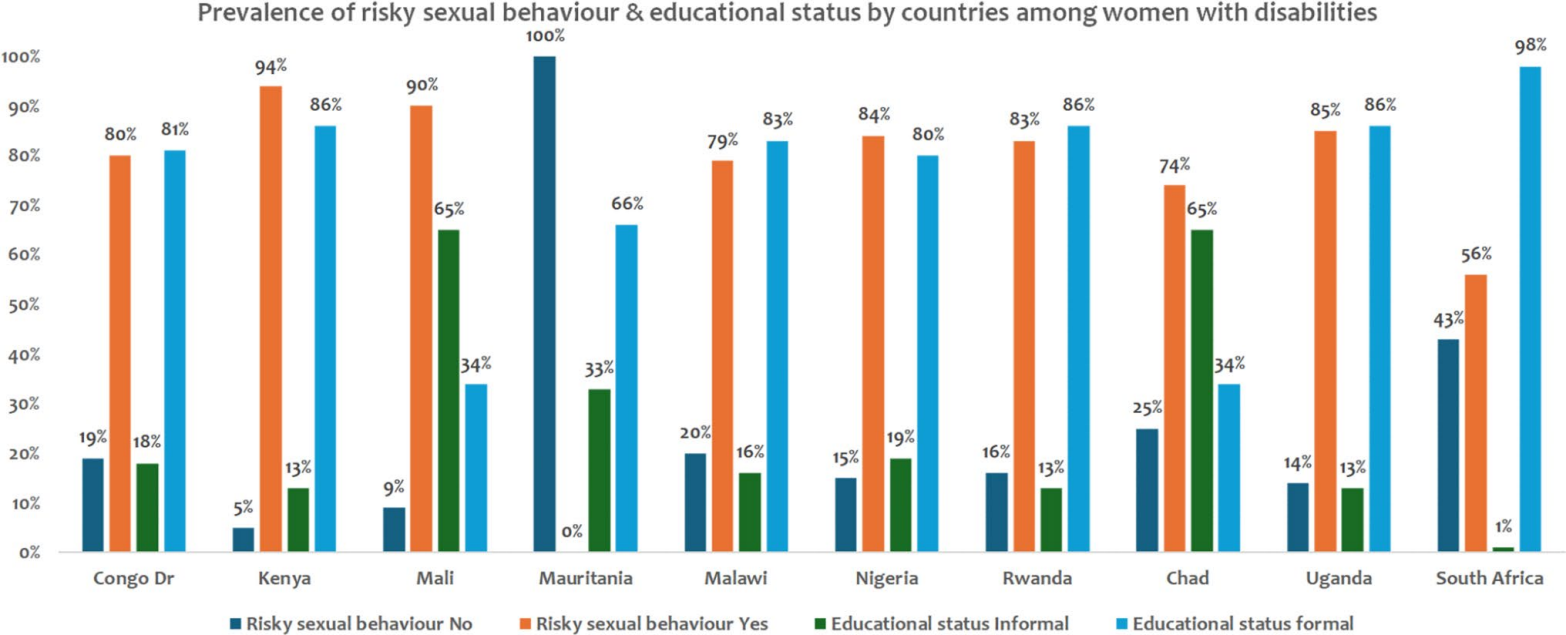
Weighted country-level prevalence of risky sexual behavior and educational status among women with disabilities in 10 Africa countries (2013–2022)

Figure 3 shows a high prevalence of risky sexual behavior among women with disabilities in countries like Kenya (94%), Uganda (85%), and Nigeria (84%), despite high levels of formal education in these settings. South Africa presents a relatively lower proportion of risky behavior (56%) alongside the highest formal education level (98%). In contrast, Mauritania records 0% risky sexual behavior and 66% formal education. Chad, Rwanda, Congo DR, and Malawi also report high levels of risky behavior, though education levels vary across these countries.

The table below shows the relationship between various factors and risky sexual behavior among women with disabilities in 10 countries in Africa. Educational status is significant, with 76.65% of formally educated women with disabilities engaging in risky sexual behavior compared to 23.35% with informal education. For age, 47.19% of women with disabilities aged 25–34 engage in risky sexual behavior, the highest among age groups. The wealth index is significantly associated with risky sexual behavior; 21.84% of the poorest women with disabilities and 18.33% of the richest women with disabilities engage in risky sexual behavior. Sexual health knowledge shows that 34.83% of women with disabilities with good knowledge levels engage in risky sexual behavior compared to 23.71% with poor knowledge. Type of residence also indicated that 73.79% of rural women with disabilities engage in risky sexual behavior compared to 26.21% of urban women with disabilities. At the community level, higher literacy (37.99% for medium literacy) and medium socioeconomic levels (7.95%) are linked to increased risky sexual behavior.

All the included explanatory variables were significant to risky sexual behavior among women with disabilities at *p *< 0.001, except mass media exposure, knowledge of contraceptive methods and community knowledge of contraceptives (Table 3).

**Table 3:** Weighted risky sexual behavior among women with disabilities by key explanatory variables

Variables (*n* = 16,517)	Frequency (%)	Risky sexual behavior		*p* value
		No	Yes	
Composite variable				
*Educational status*				<0.001
Informal education	3992 (24.71)	27.88	23.35	
Formal education	12,165 (75.29)	72.12	76.65	
Other explanatory				
*Age of respondents*				< 0.001
15–24	2506 (15.51)	15.03	15.72	
25–34	7294 (45.14)	40.35	47.19	
35 & above	6,357 (39.35)	44.63	37.09	
*Marital status*				< 0.001
Never married	976 (6.04)	9.75	4.45	
Currently married	10,277 (63.61)	59.60	65.32	
Ever married	4904 (30.35)	30.65	30.22	
*Wealth index*				< 0.001
Poorest	3600 (22.28)	23.32	21.84	
Poorer	3245 (20.09)	18.83	20.62	
Middle	3101 (19.19)	18.05	19.68	
Richer	3151 (19.50)	19.43	19.53	
Richest	3061 (18.94)	20.37	18.33	
*Mass media exposure*				0.92
No	5051 (31.26)	32.45	30.75	
Yes	11,106 (68.74)	67.55	69.25	
*Sexual health knowledge*				< 0.001
Poor	4041 (25.01)	28.05	23.71	
Moderate	6645 (41.13)	40.37	41.45	
Good	5470 (33.86)	31.58	34.83	
*Contraceptives methods knowledge*				0.47
Poor	720 (4.46)	4.71	4.35	
Moderate	67 (0.41)	0.49	0.38	
Good	15,370 (95.13)	94.80	95.27	
*Type of residence*				< 0.001
Urban	5029 (31.13)	42.62	26.21	
Rural	11,128 (68.87)	57.38	73.79	
Community levels				
*Community literacy level*				< 0.001
Low	5357 (33.15)	34.01	32.79	
Medium	5896 (36.49)	32.98	37.99	
High	4904 (30.35)	33.01	29.22	
*Community socioeconomic level*				< 0.001
Low	9985 (61.80)	63.64	61.01	
Medium	1059 (6.56)	3.30	7.95	
High	5113 (31.64)	33.06	31.04	
Community knowledge of contraceptive				0.14
Low	15,048 (93.14)	93.73	92.88	
Medium	527 (3.26)	2.29	3.68	
High	581 (3.60)	3.97	3.44	

*p *values were derived from chi-square tests

Table 4 shows the relationship between educational status and various factors among women with disabilities. 47.50% of women with disabilities aged 25–34 have formal education, compared to 37.97% with informal education. Currently, married women with disabilities with formal education account for 59.25%, while those with informal education account for 76.88%. For the wealth index, 21.99% of the richest women with disabilities have formal education compared to 9.67% with informal education. Regarding mass media exposure, 75.95% of formally educated women with disabilities are exposed compared to 46.77% with informal education.

**Table 4:** Weighted frequency and percentage of explanatory variables by educational status among women with disabilities

Variables (*n *= 16,517)	Educational status		*p* value
Explanatory variable	Informal	Formal	
	Frequency (%)	Frequency (%)	
*Age of respondents*			< 0.001
15–24	348 (8.54)	2214 (17.80)	
25–34	1550 (37.97)	5909 (47.50)	
35 & above	2184 (53.49)	4318 (34.71)	
*Marital status*			< 0.001
Never married	96 (2.35)	902 (7.25)	
Currently married	3139 (76.88)	7372 (59.25)	
Ever married	848 (20.77)	4167 (33.50)	
*Wealth index*			< 0.001
Poorest	1332 (32.64)	2349 (18.88)	
Poorer	919 (22.52)	2400 (19.29)	
Middle	817 (20.00)	2354 (18.92)	
Richer	619 (15.17	2603 (20.92)	
Richest	394 (9.67)	2736 (21.99)	
*Mass media exposure*			< 0.001
No	2173 (53.23)	2993 (24.05)	
Yes	1909 (46.77)	9449 (75.95)	
*Sexual health knowledge*			< 0.001
Poor	1570 (38.47)	2563 (20.60)	
Moderate	1599 (39.16)	5198 (41.78)	
Good	913 (22.37)	4681 (37.63)	
*Contraceptive methods knowledge*			< 0.001
Poor	610 (14.95)	126 (1.01)	
Moderate	47 (1.15)	22 (0.17)	
Good	3,425 (83.91)	12,294 (98.81)	
*Type of residence*			< 0.001
Urban	841 (20.61)	4302 (34.58)	
Rural	3241 (79.39)	8139 (65.42)	
Community levels			
*Community literacy level*			< 0.001
Low	2464 (60.36)	3014 (24.23)	
Medium	980 (24.02)	5049 (40.58)	
High	638 (15.62)	4378 (35.19)	
*Community socioeconomic level*			< 0.001
Low	3065 (75.09)	7146 (57.44)	
Medium	220 (5.39)	864 (6.94)	
High	797 (19.52)	4432 (35.62)	
*Community knowledge of contraceptives*			< 0.001
Low	3481 (85.27)	11,909 (95.72)	
Medium	350 (8.57)	189 (1.52)	
High	251 (6.16)	343 (2.76)	

*p* values were derived from chi-square tests

Additionally, 37.63% of women with disabilities with good sexual health knowledge have formal education, compared to 22.37% with informal education. Contraceptive methods knowledge shows that 98.81% of formally educated women with disabilities have good knowledge compared to 83.91% with informal education. In terms of residence, 34.58% of formally educated women with disabilities live in urban areas, compared to 20.61% of those with informal education.

Community literacy levels also show that 35.19% of women with disabilities with formal education are in high-literacy communities, compared to 15.62% with informal education. Lastly, 57.44% of women with disabilities with formal education are in low socioeconomic communities, compared to 75.09% with informal education.

All other explanatory variables were significant with the educational status of women with disabilities at *p* < 0.001.

### Multivariable Logistic Regression of Risky Sexual Behavior and Educational Status on the Explanatory Variables

Table 5 presents the results of a multivariable logistic regression analysis examining the association between risky sexual behavior and educational status among women with disabilities.

**Table 5:** Multivariable logistic regression of risky sexual behavior and educational status among women with disabilities by explanatory variables

Variable (*n* = 16,517)	Educational status	
Explanatory variable	Informal aOR [95% CI]	Formal aOR [95% CI]
*Age of respondents*		
15–24	Reference	
25–34	1.17 [0.91–1.49]	0.91 [0.81–1.03]
35 & above	0.73** [0.58–0.92]	0.64*** [0.57–0.73]
*Marital status*		
Never married	Reference	
Currently married	4.89*** [3.21–7.46]	2.83*** [2.42–3.30]
Ever married	2.73*** [1.76–4.23]	2.40*** [2.04–2.81]
*Wealth index*		
Poorest	Reference	
Poorer	1.06 [0.89–1.27]	0.98 [0.86–1.13]
Middle	0.85 [0.71–1.03]	1.03 [0.89–1.18]
Richer	1.05 [0.85–1.31]	1.14 [0.98–1.32]
Richest	0.78 [0.56–1.09]	1.04 [0.86–1.27]
*Mass media exposure*		
No	Reference	
Yes	1.14 [0.99–1.31]	1.12* [1.01–1.25]
*Sexual health knowledge*		
Poor	Reference	
Moderate	1.27** [1.09–1.48]	1.17** [1.05–1.31]
Good	1.28** [1.07–1.52]	1.44*** [1.28–1.61]
*Contraceptives methods knowledge*		
Poor	Reference	
Moderate	1.48 [0.69–3.16]	o.43 [0.15–1.22]
Good	0.79* [0.65–0.96]	1.86** [1.27–2.72]
*Type of residence*		
Urban	Reference	
Rural	1.64*** [1.36–1.99]	3.22*** [2.89–3.60]
Community levels		
*Community literacy level*		
Low	Reference	
Medium	0.72*** [0.61–0.85]	1.10 [0.99–1.23]
High	0.61*** [0.50–0.75]	0.65*** [0.58–0.73]
*Community socioeconomic level*		
Low	Reference	
Medium	3.38*** [2.34–4.87]	2.20*** [1.80–2.68]
High	1.62*** [1.26–2.09]	1.73*** [1.51–1.99]
*Community knowledge of contraceptive*		
Low	Reference	
Medium	1.58*** [1.23–2.02]	1.86** [1.27–2.72]
High	1.68** [1.23–2.32]	1.42* [1.05–1.92]
Pseudo-*R*^*2*^	0.05	0.07

*p < 0.05, **p < 0.01, ***p < 0.001

Age of respondents shows that women with disabilities aged 35 and above were significantly less likely to engage in risky sexual behavior compared to those aged 15–24, both in informal [aOR = 0.73**; 95%(CI 0.58–0.92)] and formal education groups [aOR = 0.64**; 95%(CI 0.57–0.73)] while marital status shows that currently married women with disabilities were significantly more likely to engage in risky sexual behavior compared to never-married women with disabilities, with higher odds in the informal education group [aOR = 4.89***; 95%(CI 3.21–7.46)] than in the formal education group [aOR = 2.83**; 95%(CI 2.24–3.30)].

Mass media exposure showed an association with increased odds of risky sexual behavior in the formal education group [aOR = 1.12*; 95%(CI 1.01–1.25)] but not significantly in the informal education group. Sexual health knowledge shows that good sexual health knowledge was associated with higher odds of risky sexual behavior in both educational groups, with aORs ranging from 1.17 to 1.44 and significant *p* values (*p *< 0.01 and *p * < 0.001).

Women with disabilities who reside in medium and high community socioeconomic levels were significantly associated with increased odds of risky sexual behavior in both educational groups, with aORs ranging from 1.62 to 3.38 and* p* values less than 0.001.

The model fit pseudo-*R*^2^ values indicate that the model explains 5% of the variance in risky sexual behavior for women with disabilities with informal education and 7% for those with formal education among women with disabilities in 10 African countries.

### Multivariate Decomposition of Disparity in Risky Sexual Behavior Between Informal and Formal Education

The majority of the disparity (81.93%) in risky sexual behavior between women with disabilities with informal and formal education was due to differences in coefficients (C), while 18.07% was attributed to differences in characteristics (E). Among significant findings, women with disabilities aged 35 and above contribute 2.25% to the disparity due to characteristics, while their coefficients show a negative contribution of − 0.92%. Currently, married women with disabilities significantly influence the disparity, contributing − 2.47% due to characteristics and − 2.33% due to coefficients, indicating a lower likelihood of risky behavior compared to never-married women with disabilities. Good sexual health knowledge contributes significantly, with 2.20% due to characteristics and 1.13% due to coefficients. Good knowledge of contraceptive methods shows a substantial positive impact on the disparity, contributing 4.41% due to coefficients. Rural residence is another significant factor, contributing − 2.45% due to characteristics but a higher 6.10% due to coefficients. Community literacy levels show a medium-level contribution of 3.91% due to coefficients and high levels contributing − 2.46% due to characteristics. Medium socioeconomic levels contribute 2.35% due to characteristics and − 1.91% due to coefficients, while high socioeconomic levels add 2.38% due to characteristics. Finally, medium community knowledge of contraceptives contributes − 2.02% due to characteristics (Table 6).

**Table 6:** Multivariate decomposition of associated factors with risky sexual behavior disparity between informal and formal education

Variable (*n* = 16,517)	Difference due to characteristics (E)		Difference due to coefficient (C)	
Characteristics	Coefficient	Percentage (%)	Coefficient	Percentage (%)
The total disparity percentage explained		18.07		81.93
*Age of respondents*				
15–24	Reference		Reference	
25–34	− 0.00139	− 1.30	− 0.01733	− 1.78
35 & above	0.01190*	2.25	− 0.01240	− 0.92
*Marital status*				
Never married	Reference		Reference	
Currently married	− 0.02506*	− 2.47	− 0.07949*	− 2.33
Ever married	0.01451*	2.41	− 0.00506	− 0.54
*Wealth index*				
Poorest	Reference		Reference	
Poorer	0.00002	0.22	− 0.00288	− 0.65
Middle	− 0.0000	− 0.38	0.00689	1.55
Richer	0.00090	1.36	0.00219	0.55
Richest	0.00077	0.43	0.00451	1.46
*Mass media exposure*				
No	Reference		Reference	
Yes	0.00531	1.56	− 0.00096	− 0.13
*Sexual health knowledge*				
Poor	Reference		Reference	
Moderate	0.0072	1.79	− 0.00565	− 0.78
Good	0.00691*	2.20	0.00539	1.13
*Contraceptives methods knowledge*				
Poor	Reference			
Moderate	0.00122	1.45	− 0.00264	− 1.82
Good	0.01560	1.44	0.13173***	4.41
*Type of residence*	Reference		Reference	
Urban				
Rural	− 0.01943*	− 2.45	0.09808***	6.10
Community levels				
*Community literacy level*				
Low	Reference		Reference	
Medium	0.00254	1.34	0.01793***	3.91
High	− 0.01191*	− 2.46	0.00166	0.46
*Community socioeconomic level*				
Low	Reference		Reference	
Medium	0.00179*	2.35	− 0.00440	− 1.91
High	0.01191*	2.38	0.00231	0.45
*Community knowledge of contraceptive*				
Low	Reference		Reference	
Medium	− 0.00775*	− 2.02	0.00302	0.71
High	− 0.00172	− 1.72	− 0.00174	− 0.77

*p < 0.05, **p < 0.01, ***p < 0.001

## Discussion

In this study, we found a high prevalence of risky sexual behavior among women with disabilities across the countries surveyed (ranging from 56% in South Africa to 94% in Kenya), except for Mauritania, where a prevalence of 0% was recorded. Also, we found varied inter-country levels of risky sexual behavior, with higher prevalence observed in the southern and central parts of most of the countries surveyed, especially in Chad, Malawi, Rwanda, the Democratic Republic of Congo, Uganda, and South Africa. Factors that significantly contributed to the variations in risky sexual behavior between women with disabilities with formal and informal education include age, marital status, sexual health knowledge, community socioeconomic levels, and mass media exposure.

Although there are limited comparable prevalence estimates of risky sexual behavior among women with disabilities for most of the countries in the present study, the findings show a higher prevalence of risky sexual behavior than previously reported among women with disabilities in countries like Uganda (17.1%) (Bukuluki et al., 2023), and Burkina Faso (52.3%) (Cissé et al., 2022). Perhaps, the disparities in settings, definition of risky sexual behavior, and time gap contributed to the variations in prevalence. Nonetheless, the current findings highlight the increased exposure of women with disabilities in Africa to sexual and reproductive health problems, including HIV infection, due to high levels of risky sexual behavior (De-Beaudrap et al., 2014; Groce et al., 2009). Meanwhile, we found varied inter-country levels of risky sexual behavior, with higher proportions observed in the southern and central parts of most of the countries surveyed. While the reasons for the inter-country level variations remain unclear, the finding suggests the need for public health planning and interventions aimed at reducing risky sexual behaviors among women with disabilities to be targeted at areas with high proportions, particularly in the southern and central parts of Chad, Malawi, and Rwanda. Interestingly, Mauritania recorded a 0% prevalence of risky sexual behavior, which calls for further studies to ascertain the level of risky sexual behavior among women with disabilities in the country. This is also particularly important because no studies have been conducted specifically on risky sexual behavior even among women without disabilities which be used to compared but studies with measurement relating to risky sexual behavior has reported such as transactional sex and other sexual vices (Lardoux & N’Bouke, 2013; Olusegun Babaniyi, 2015).

The findings revealed that women with disabilities aged 35 years and above were significantly less likely to engage in risky sexual behavior compared to those aged 15–24, in both women with formal and informal education. The current findings agree with findings from previous studies in Ethiopia (Kassa et al., 2016) and Burkina Faso (Cissé et al., 2022), which reported that age has a significant impact on the risky sexual behavior of women with disabilities, with younger women having the greatest risk. Besides, evidence shows that young women have increased exposure to unsafe sexual practices, including high frequency of condomless sex, in both with (Baines et al., 2018) and without disabilities (Baru et al., 2020; Hlongwa et al., 2020) populations. The increased exposure of young women with disabilities to risky sexual behavior has been associated with a lack of sexual education, social isolation (Matin et al., 2021), peer pressure, substance use (Sifer & Getachew, 2024), and sexual violence and abuse (Bolarinwa et al., 2025a). Meanwhile, despite their increased exposure to risky sexual behavior, women with disabilities have limited access to sexual and reproductive health services (Kassa et al., 2016), including access to contraceptives like condoms (Horner-Johnson et al., 2019). Therefore, the current findings emphasize the importance of age-targeted interventions that empower young women with disabilities to assert their sexual and reproductive rights and improve access to sexual and reproductive health services to reduce their exposure to risky sexual behaviors.

Contrary to findings from previous studies that reported no significant relationship between marital status and sexual risk behavior among women with disabilities (Kwagala & Galande, 2022; Seidu et al., 2021), our findings revealed that currently married women were more likely to engage in risky sexual behavior relative to never-married women with disabilities, with those with informal education having about a twofold increase in risk than those with formal education. Perhaps, the differences in the scope of the definition of risky sexual behavior in the present study (condomless sex and multiple sexual partners) relative to the previous studies might have accounted for the observed disparities. For instance, Anglewicz and Clark (2013) reported that married women are more likely to engage in condomless sex because they have a regular sexual partner. Meanwhile, the highly risky sexual behavior observed among married women with disabilities with informal education could be explained by the influence of education on sexual risk behavior, as evidence suggests that lack of formal education has a negative impact on women’s autonomy and decision-making capacity to negotiate for safer sex (Seidu et al., 2021).

Surprisingly, we found that having good sexual health knowledge was associated with higher odds of risky sexual behavior in both disabled women with formal and informal education. The current findings contradict previous studies, which suggested that having good sexual health knowledge reduces women’s exposure to risky sexual behaviors (Bukuluki et al., 2023; Richner & Lynch, 2024). Meanwhile, in South Africa, Rohleder et al. (2012) reported that most individuals with disabilities engaged in condomless sex, despite having knowledge of the importance of condoms in the prevention of HIV infection. Plausibly, despite having good sexual health knowledge, most of the women with disabilities may be lacking the capacity to engage in safer sex practices due to limited autonomy in sexual health decision-making (Perez-Curiel et al., 2023), and fear of sexual victimization or rejection by their partners (Linton et al., 2016; Touko et al., 2010). Therefore, aside from providing adequate sexual health knowledge, it is important to implement measures that build the capacity of women with disabilities to exercise control over their sexual behavior (Rohleder et al., 2012) and promote their sexual self-efficacy and autonomy (Richner & Lynch, 2024), to reduce exposure to risky sexual behaviors among the women with disabilities.

Meanwhile, available evidence shows that poverty often drives women with disabilities into risky sexual behaviors, including having multiple sexual partners, engaging in condomless sex and coerced sexual intercourse, to obtain financial and material support for survival (Nyindo, 2005; Tarkang et al., 2015). While the current findings showed no significant relationship between wealth index and risky sexual behavior among the women with disabilities, we observed that those who resided in communities with medium and high socioeconomic levels had increased odds of risky sexual behavior in both those with formal and informal education. Perhaps, because individuals in communities with high socioeconomic levels are likely to be socioeconomically advantaged and may be providing material and financial support to women with disabilities, they may be taking undue sexual advantage of the women with disabilities thus predisposing them to risky sexual behaviors. Considering that women with disabilities often do not seek help when they are sexually exploited or abused due to fear of victimization (Hunt et al., 2023), especially if the perpetrator is a benefactor, there is a need for increased attention and interventions to prevent sexual exploitation and abuse of women with disabilities who reside in communities with high socioeconomic status in Africa.

Although previous studies reported positive associations between mass media exposure and safer sex practices among women (Aboagye et al., 2021; Bessinger et al., 2004), the current findings revealed that exposure to mass media increased the odds of risky sexual behavior among women with disabilities with formal education, but not among those with informal education. Arguably, compared to women with disabilities with informal education, those with formal education are more likely to have increased access to and utilization of various forms of media. While education may improve access and use of mass media among women with disabilities, the women may also be exposed to harmful media content, including inappropriate sexual media (Carpentier et al., 2007), which could increase their propensity to engage in risky sexual behavior. Therefore, there is a need to consider the usefulness of mass media in promoting safer sex practices (Aboagye et al., 2021). Policymakers need to implement strategies that promote media literacy and limit the exposure of women with disabilities to sexually harmful media content.

### Strengths and Limitations

The current study used the most recent nationally representative DHS datasets of 10 countries in Africa. Therefore, the findings could be generalized to the women with disabilities in various countries. Despite these strengths, the study has some limitations. First, although risky sexual behavior encompasses multiple behaviors, including engaging in sexual intercourse with a less-known partner, early sexual debut, condomless sex, multiple sexual partners, and casual sex among others (Alemu & Fantahun, 2011; Cissé et al., 2022), the phenomenon was operationalized in the present study as having multiple sexual partners or engaging in condomless sex, which needs be considered when interpreting our findings. Second, the current study did not segregate respondents by type of disability which could limit the interpretation of our findings, since type of disability could influence risky sexual behavior among the women (Bolarinwa et al., 2025b; Parekh et al., 2023). Also, the current findings only reported on the associations between the variables and could not draw causal inferences, due to the use of cross-sectional survey design.

### Policy and Practical Implications

The findings of this study are important in developing policy and effective strategies to address the issue of risky sexual behavior among women with disabilities in Africa. First, given the high prevalence of risky sexual behavior among both women with disabilities with formal education and informal education, there is a need for public health planning and interventions that increase awareness of sexual risk behaviors among women with disabilities and promote safer sex practices like consistent condom use. Special attention must be given to areas with a high prevalence of risky sexual behaviors, particularly in the southern and central parts of countries like Chad, Malawi, and Rwanda. Also, the interventions to prevent risky sexual behavior among women with disabilities should be targeted at younger women with disabilities, those who are married, those with limited sexual health knowledge, those with increased exposure to mass media, and those living in communities with medium and high socioeconomic levels. For instance, empowering young women with disabilities through sexual health education and skills acquisition could enable them to assert their sexual and reproductive rights and thereby minimize their exposure to risky sexual behavior. Given the lack of prevalence estimates of risky sexual behavior among women with disabilities in most of the countries studied, the current prevalence findings could serve as baseline data for future studies and monitoring trends.

### Conclusion and Recommendations

The study findings show that women with disabilities with formal education, as well as those with informal education, are highly exposed to risky sexual behavior in Africa. Therefore, there is a need for targeted interventions that minimize risky sexual behavior among women with disabilities in those countries. Such interventions could be targeted at young women with disabilities, those who are married, those with limited sexual health knowledge, those with increased exposure to mass media, and those living in communities with medium and high socioeconomic levels. Special attention must be given to areas with a high prevalence of risky sexual behaviors, particularly in the southern and central parts of countries like Chad, Malawi, and Rwanda. Implementing these measures could reduce the exposure of women with disabilities to risky sexual behaviors across Africa and thereby minimize their risk for adverse sexual and reproductive health outcomes.

## Data Availability

The datasets utilized in this study can be accessed at https://dhsprogram.com/data/available-datasets.cfm.
